# The Therapeutic effect of Memantine through the Stimulation of Synapse Formation and Dendritic Spine Maturation in Autism and Fragile X Syndrome

**DOI:** 10.1371/journal.pone.0036981

**Published:** 2012-05-15

**Authors:** Hongen Wei, Carl Dobkin, Ashfaq M. Sheikh, Mazhar Malik, W. Ted Brown, Xiaohong Li

**Affiliations:** 1 Department of Neurochemistry, New York State Institute for Basic Research in Developmental Disabilities, New York, New York, United States of America; 2 Department of Human Genetics, New York State Institute for Basic Research in Developmental Disabilities, New York, New York, United States of America; 3 Shanghai Mental Health Center, Shanghai Jiao Tong University School of Medicine, Shanghai, China; University of Edinburgh, United Kingdom

## Abstract

Although the pathogenic mechanisms that underlie autism are not well understood, there is evidence showing that metabotropic and ionotropic glutamate receptors are hyper-stimulated and the GABAergic system is hypo-stimulated in autism. Memantine is an uncompetitive antagonist of NMDA receptors and is widely prescribed for treatment of Alzheimer's disease treatment. Recently, it has been shown to improve language function, social behavior, and self-stimulatory behaviors of some autistic subjects. However the mechanism by which memantine exerts its effect remains to be elucidated. In this study, we used cultured cerebellar granule cells (CGCs) from *Fmr1* knockout (KO) mice, a mouse model for fragile X syndrome (FXS) and syndromic autism, to examine the effects of memantine on dendritic spine development and synapse formation. Our results show that the maturation of dendritic spines is delayed in *Fmr1*-KO CGCs. We also detected reduced excitatory synapse formation in *Fmr1*-KO CGCs. Memantine treatment of *Fmr1*-KO CGCs promoted cell adhesion properties. Memantine also stimulated the development of mushroom-shaped mature dendritic spines and restored dendritic spine to normal levels in *Fmr1*-KO CGCs. Furthermore, we demonstrated that memantine treatment promoted synapse formation and restored the excitatory synapses to a normal range in *Fmr1*-KO CGCs. These findings suggest that memantine may exert its therapeutic capacity through a stimulatory effect on dendritic spine maturation and excitatory synapse formation, as well as promoting adhesion of CGCs.

## Introduction

Autism is the most severe of a group of neurodevelopmental disorders referred to autism spectrum disorders (ASDs), and is characterized by problems in communication, social skills, and repetitive behavior. Susceptibility to autism is attributable to genetic factors [1], [2], [3], [4], but the exact cause of this disorder is not yet known. There is some evidence showing that metabotropic and ionotropic glutamate receptors are affected in autism. Purcell et al [5] demonstrated that there is excessive glutaminergic activity in autistic brain. A normal level of glutamate is important for neurotransmitters that play a key role in long-term potentiation, learning and memory [6], [7], [8], [9]. Hypo-function of the inhibitory GABAergic system and hyperactivity of the excitatory glutamate system has been theorized to have a causal role in autism [10].

**Figure 1:**
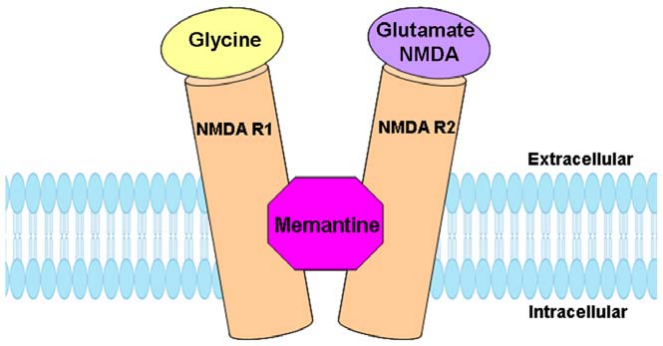
Schematic representation of memantine and NMDA receptor.

Memantine is a low-affinity voltage-dependant uncompetitive antagonist at NMDA receptors [11], [12], as shown in Figure 1. It is widely prescribed for the treatment of Alzheimer's disease (AD). Studies have shown that memantine can be rapidly displaced from the NMDA receptor, which may avoid prolonged receptor blockade and the associated detrimental effects on learning and memory [13]. In addition, memantine has been demonstrated to act as an antagonist at nicotinic acetylcholine receptors and at 5-HT receptors [14], [15]. Both *in vitro* and *in vivo* studies indicate that memantine offers protective effects from neurotoxicity and can improve learning and memory in several preclinical models of AD [16], [17], [18], [19]. Perry et al [20] reported decreased nicotinic receptors and decreased cholinergic receptors in the cortices of autistic brain. Several studies have reported beneficial results with the utilization of cholinesterase inhibitors in AD to enhance frontal lobe function and promote executive language and social response in autism [21], [22], [23]. Most recently, two small open-label studies reported the effect of memantine on improving language and behavior of autistic subjects [24], [25]. In a large scale clinical trial study involving 151 subjects, Chez et al [26] further observed that open-label use of memantine significantly improved language function, social behavior, and self-stimulatory behaviors of autistic subjects. Studies are underway with memantine and larger numbers of autistic subjects (Clinicaltrials.gov). In addition, Erickson et al [27] reported that 4 of 6 patients with Fragile X syndrome (FXS) and a co-morbid diagnosis of pervasive developmental disorder (PDD) with memantine treatment showed global clinical benefit on ratings with the Clinical Global Impressions-Improvement subscale (CGI-I). However the cellular mechanism by which memantine improves language and social behaviors in autism remains to be elucidated.

In this study, we examined the effects of memantine on the cultured cerebellar granule cells (CGCs) from *Fmr1* knockout (*Fmr1*-KO) mice, which is a mouse model for FXS and currently also used as a mouse model for autism studies [28], [29], [30]. Our results show that the maturation of dendritic spines is delayed in *Fmr1*-KO CGCs. We also detected reduced excitatory synapse formation in *Fmr1*-KO CGCs. Memantine treatment of *Fmr1*-KO CGCs promoted cell adhesion properties. Memantine also stimulated the development of mushroom-shaped dendritic spines and restored dendritic spine to normal levels seen in *Fmr1*-KO CGCs. Furthermore, we demonstrate that memantine treatment promoted synapse formation and restored the excitatory synapses to a normal range in *Fmr1*-KO CGCs.

## Methods

### *Fmr1* knockout mice

Mice were from a colony of congenic C57BL/6 fragile X mice derived from C57BL/6-129 hybrid mice carrying the *Fmr1* knockout mutation originally provided through the generosity of B. Oostra, P.Willems and S.Warren. Offspring of mice that carried the knockout mutation were distinguished from their normal siblings by polymerase chain reaction (PCR) analysis with 1.5 mM MgCl_2_ and using S1, S2 primers for the normal allele and M2, N2 primers for the knockout allele essentially as originally described [28]. All experiments with live animals conformed to the guidelines for the ethical use of animals of the NYS Institute for Basic Research in Developmental Disabilities Institutional Animal Care and Use Committee (IACUC).

### Cell culture and memantine treatment

CGCs were prepared from wild type (WT) and *Fmr1* KO 5–6 day postnatal pups as described previously [31]. Briefly, the entire cerebellum was dissected out, and single cell suspensions were prepared by trypsinization and trituration in 1% trypsin in Ca^2+^-free isotonic phosphate buffer (CF-PBS). Cells were washed in CF-PBS and resuspended in culture medium (MEM), supplemented with 0.25% glucose, 2 mM glutamine, 10% HS, 5% FCS, and both penicillin and streptomycin. Cells were seeded into poly-D-lysine-coated dishes and incubated at 37°C in a moist chamber under 5% CO_2_. After 24 hr *in vitro* the medium was replaced with serum-free medium containing 15% N-2 supplement and 15 mM KCl in the medium. After 5–7 days *in vitro* (DIV) CGCs were used for the experiments reported in the present study. When required, the CGCs were treated for 72 h with memantine (Sun Pharma) at a concentration of 100 µM, referred from the literature [32], [33].

### Cell adhesion assay

Approximately 10,000 CGCs were plated per well in 96 well tissue culture plates coated with poly-D-lysine (Sigma-Aldrich) at a final concentration of 10 µg/ml. After 1.5 h of attachment, unattached cells were removed by aspiration and adherent cells were quantified by the colorimetric aqueous MTS assay (CellTiter 96 AQ_ueous_ One Solution kit, Promega).

### Cell migration assay

CGCs were labeled with fluorescent Calcein AM (BD Biosciences) at a final concentration of 2.5 µM. Approximately 10,000 labeled cells were plated in 0.8 ml DMEM +1% FBS in each well of a 24 well chamber plate adapted for the HTS Fluoroblock (BD Falcon) apparatus. After 3 h, 5% FBS was added to the lower chamber medium to establish a 1–5% serum gradient and migration of cells from the upper to lower chamber were quantified at 2 h using a microfluorimetric plate reader (CytoFluor 4000, MTX Lab Systems) [34].

### DiI labeling

The culture was labeled using a protocol adapted from Hering *et al* [35] Briefly, CGCs were fixed in 4% formaldehyde for 15 min and incubated with Vybrant-DiI cell-labeling solution (1∶200, Invitrogen) for 25 min at 37°C. Cultures were washed in warmed PBS, incubated in PBS at 4°C for 24–48 hr to allow dye diffusion within membranes, mounted on glass slides with ProLong Gold antifade reagent (Invitrogen), and then imaged using a Nikon Eclipse E800 microscope. Spines were classified according to previously described criteria [36], [37]: the mushroom type has a large head with short neck; the stubby type has a short protrusion with no clear neck; the thin type has a long neck and a small head. Spine length was measured from shaft to tip using a bent-line tool [38]. The spines were counted in 12–18 neurons/group from the independent experiments. For each neuron, 1–4 dendrites were analyzed. The spine value was averaged in each independent experiment for the statistical test. The n value refers to the number of independent experiments analyzed.

### Immunofluorescence

CGCs were fixed in 4% formaldehyde for 15 min and blocked with 3% goat serum/0.3% Triton X-100 in PBS and incubated with an anti-Syp polyclonal antibody (anti-synaptophysin, 1∶200, Cell Signaling Technology), anti-VGLUT1 monoclonal antibody (1∶500, Millipore), and anti-VGAT polyclonal antibody (1∶500, Millipore) overnight at 4°C, followed by incubation with Alexa Fluor 488 anti-rabbit IgG and Alexa Fluor 555 anti-mouse IgG (1∶1000, Invitrogen) for 1.5 h at room temperature. Cell number and distribution were analyzed following staining with nuclear marker Hoechst 33258 (Sigma-Aldrich) for 5 min at room temperature. Cultures were mounted on glass slides with ProLong Gold antifade reagent (Invitrogen), and imaged using a Nikon Eclipse E800 microscope.

The analysis of immunofluorescent images was done as described previously [39]. Images were acquired in the linear range with constant settings and analyzed using Image J software (National Institutes of Health, Bethesda, MD, USA). All analyses were performed blind as to the treatment of the culture. Immunoreactive puncta were defined as discrete regions along the dendrite with fluorescence intensity twice the background and average size of the puncta were normalized with data from the WT CGCs group, respectively. For quantification, 20–30 neurons from two to three different batches of cultures and experiments for each condition were randomly chosen on the basis of having a healthy morphology. Negative controls, in which the primary antibodies were omitted and treated only with the secondary antibodies, were run for each condition to exclude false positive secondary antibody binding. The n value refers to the number of cells analyzed.

### Statistics

The statistical analyses were carried out by one-way analysis of variance (ANOVA) with Fisher's PLSD post hoc test using the StatView 5.0 software (SAS Institute, Inc.). The Kolmogorov-Smirnov test was used to compare the two distributions. All data is presented as means ± SE. Significance was accepted at p<0.05 or better.

## Results

### Memantine promotes CGCs adhesion

**Figure 2:**
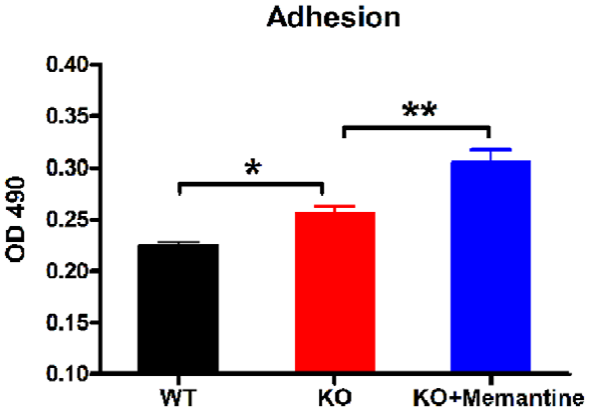
Memantine increased the adhesion property in *Fmr1*-KO CGCs. Comparison of CGCs adhesion. The values are mean ±SE from three independent experiments with five replicates in each experiment, ^*^p<0.05, ^**^p<0.01. OD: optical density.

To test the effect of memantine on adhesion of CGCs, *Fmr1*-KO and WT CGCs were seeded on the PDL-coated surface. Cell adhesion was evaluated by a modified MTS assay based on dehydrogenase conversion of MTS to colored tetrazolium salt, a reaction that is mediated only by viable cells. The amount of colored product formed at 490 nm (OD 490) was proportional to the number of attached cells. As shown in Figure 2, there was an increase in cell adhesion in *Fmr1*-KO CGCs compared to WT CGCs (one-way ANOVA, F = 22.42, p<0.0001; Fisher's PLSD, p = 0.03), and after treatment with memantine, *Fmr1*-KO CGCs exhibited a 19% increase in adhesion (Fisher's PLSD, p = 0.002).

### Memantine has little effect on CGCs migration

**Figure 3:**
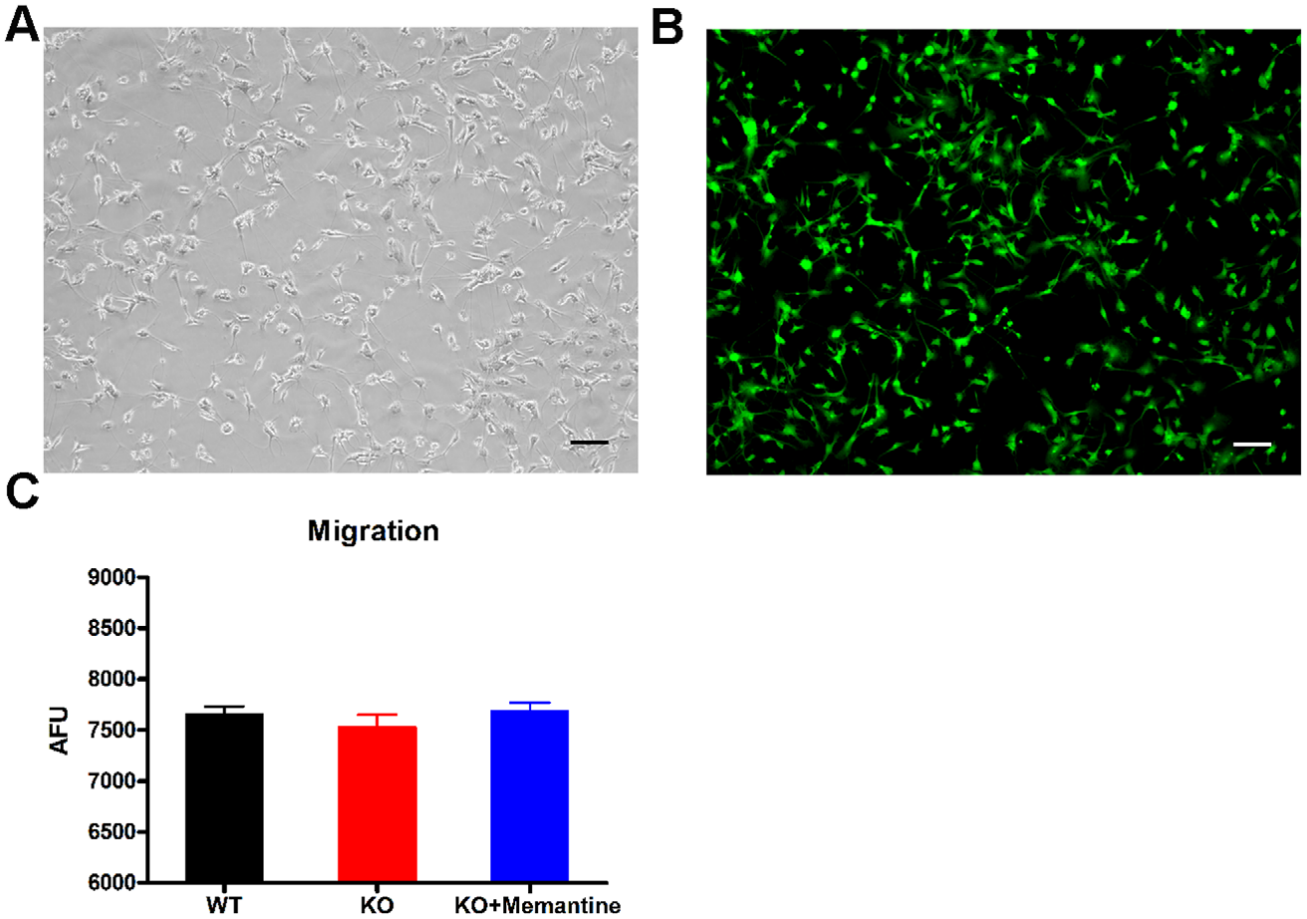
Memantine had little effect on CGCs migration. A. Cultured CGCs. Scale bar, 100 µm. B. CGCs stained with Calcein AM dye in modified Boyden chamber migration assay. Scale bar, 100 µm. C. Comparison of CGCs migration. The values are mean ±SE from three independent experiments with five replicates in each experiment. AFU: arbitrary fluorescence units.

Cell migration is modulated by a complex of adhesion molecules interacting between migrating cells and the surrounding extracellular matrix proteins, and is critically affected by cell adhesion [40]. Since we observed that memantine promotes CGC adhesion, we further investigated whether memantine affects CGC migration with a modified Boyden chamber assay [34]. This assay enabled us to count the cells migrating through the insert into lower chamber. Our results indicated that memantine has little effect on CGCs migration as we observed no significant difference in cell migration in comparing WT CGCs, *Fmr1*-KO CGCs and *Fmr1*-KO CGCs treated with memantine (one-way ANOVA, F = 0.70, p = 0.52, Figure 3).

### Memantine treatment restores dendritic spine to normal levels in *Fmr1*-KO CGCs

**Figure 4:**
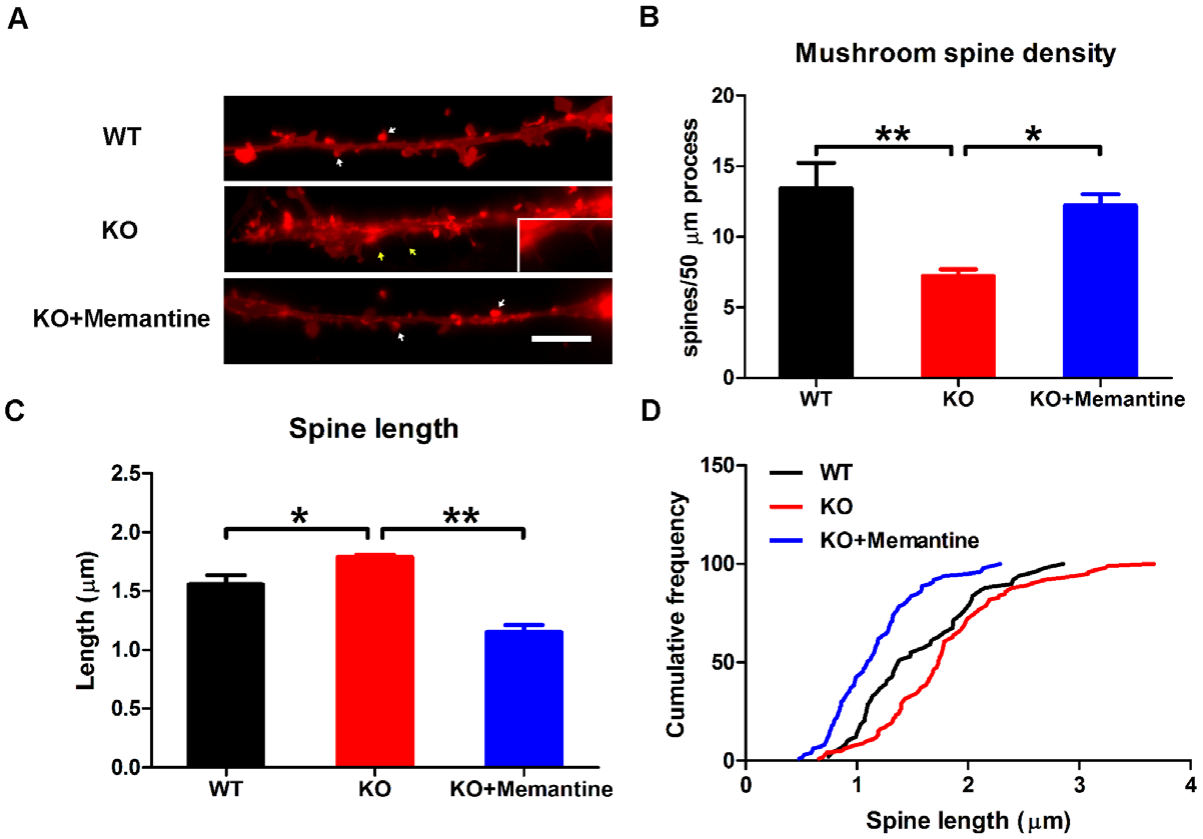
Memantine restored dendritic spine to normal levels in *Fmr1*-KO CGCs. A. Cultured CGCs were stained with Vybrant-DiI to outline the shape of dendritic spines. Arrows colored in white show the mushroom-shaped spines, while arrows colored in yellow show the thin-shaped spines, Scale bars, 10 µm. Histogram showed the density of mushroom-shaped spines (B), spine length (C) and cumulative frequency distribution of spine lengths (D) in WT CGCs, *Fmr1*-KO CGCs and *Fmr1*-KO CGCs treated with memantine. The data are the mean ± SE, ^*^p<0.05, ^**^p<0.01. n = 3.

Dendritic spine dynamics plays an important role in mediating learning and memory, and is of essential importance to synaptic function [41], [42]. To test whether memantine treatment affected the development of dendritic spines, DiI labeling was employed to outline the shape of dendritic spines (Figure 4A). Our results showed that the density of mushroom-shaped mature dendritic spines was significantly decreased in *Fmr1*-KO CGCs as compared with WT CGCs (one-way ANOVA, F = 7.86, p = 0.02; Fisher's PLSD, p<0.01, Figure 4B). However, treatment with memantine stimulated the development of dendritic spines and restored the density of mushroom-shaped spines in *Fmr1*-KO CGCs to the normal level (Fisher's PLSD, p = 0.02, Figure 4B). We examined the effects of memantine on spine length and found that dendritic spines were longer in *Fmr1*-KO CGCs than in WT CGCs (one-way ANOVA, F = 31.43, p<0.0001; Fisher's PLSD, p = 0.03). Memantine decreased the spine length in *Fmr1*-KO CGCs (Fisher's PLSD, p<0.0001, Figure 4C). In addition, the distribution of different length of spines was altered after memantine treatment (Figure 4D). *Fmr1*-KO CGCs exhibited fewer short spines and more medium to long spines (Kolmogorov-Smirnov Comparison, p = 0.01), while more short spines were present in memantine-treated *Fmr1*-KO CGCs as compared with *Fmr1*-KO CGCs without treatment (Kolmogorov-Smirnov Comparison, p<0.0001).

### Memantine stimulates synapse formation and restores the excitatory synapses to a normal range in KO CGCs

**Figure 5:**
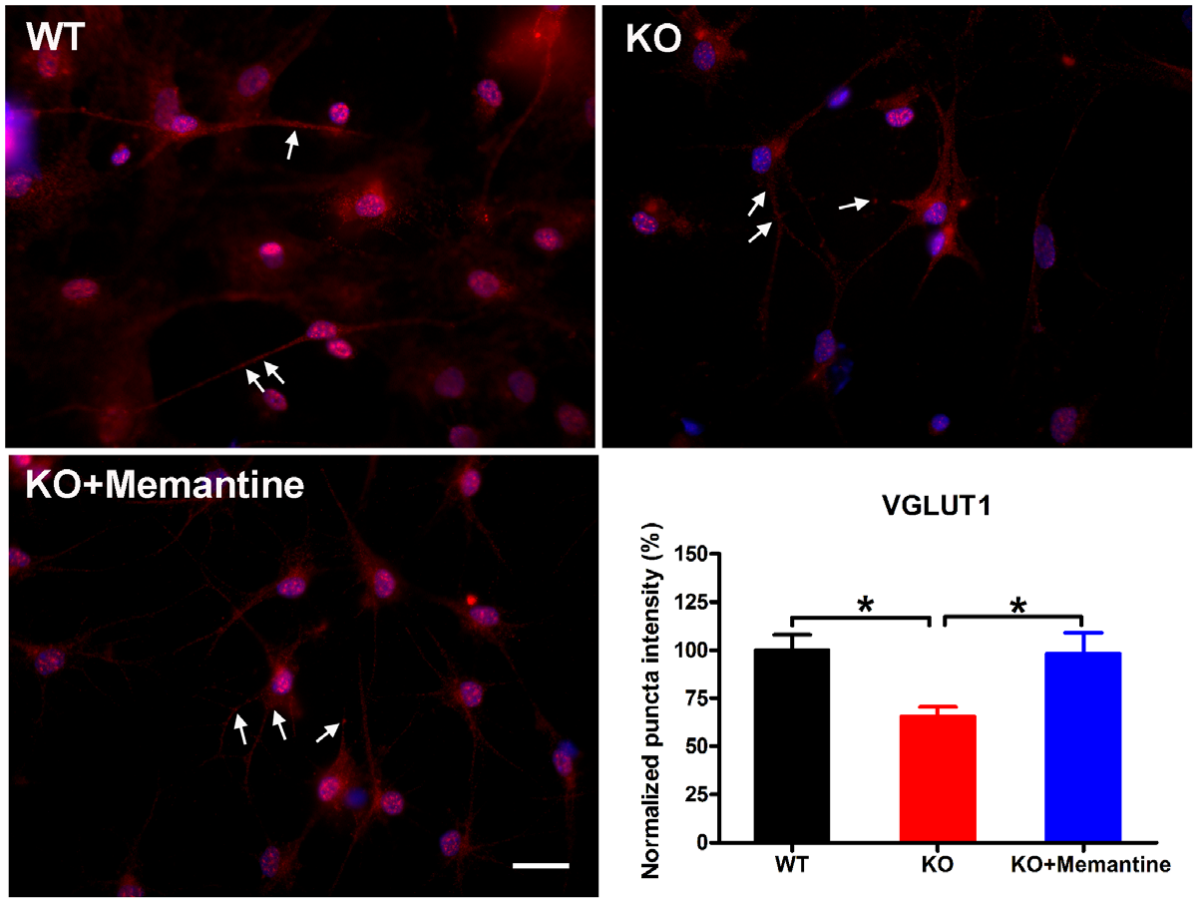
Memantine fully restored the intensity of excitatory synapses to a normal range in *Fmr1*-KO CGCs. Representative photomicrographs of labeling of VGLUT1 (arrow) in WT CGCs, *Fmr1*-KO CGCs and *Fmr1*-KO CGCs treated with memantine. Red, VGLUT1; Blue, Hoechst, scale bars, 25 µm. The histogram shows quantification of puncta size using image analysis. WT, n = 23; *Fmr1*-KO, n = 25; *Fmr1*-KO+Memantine, n = 26. ^*^p<0.05.

**Figure 6:**
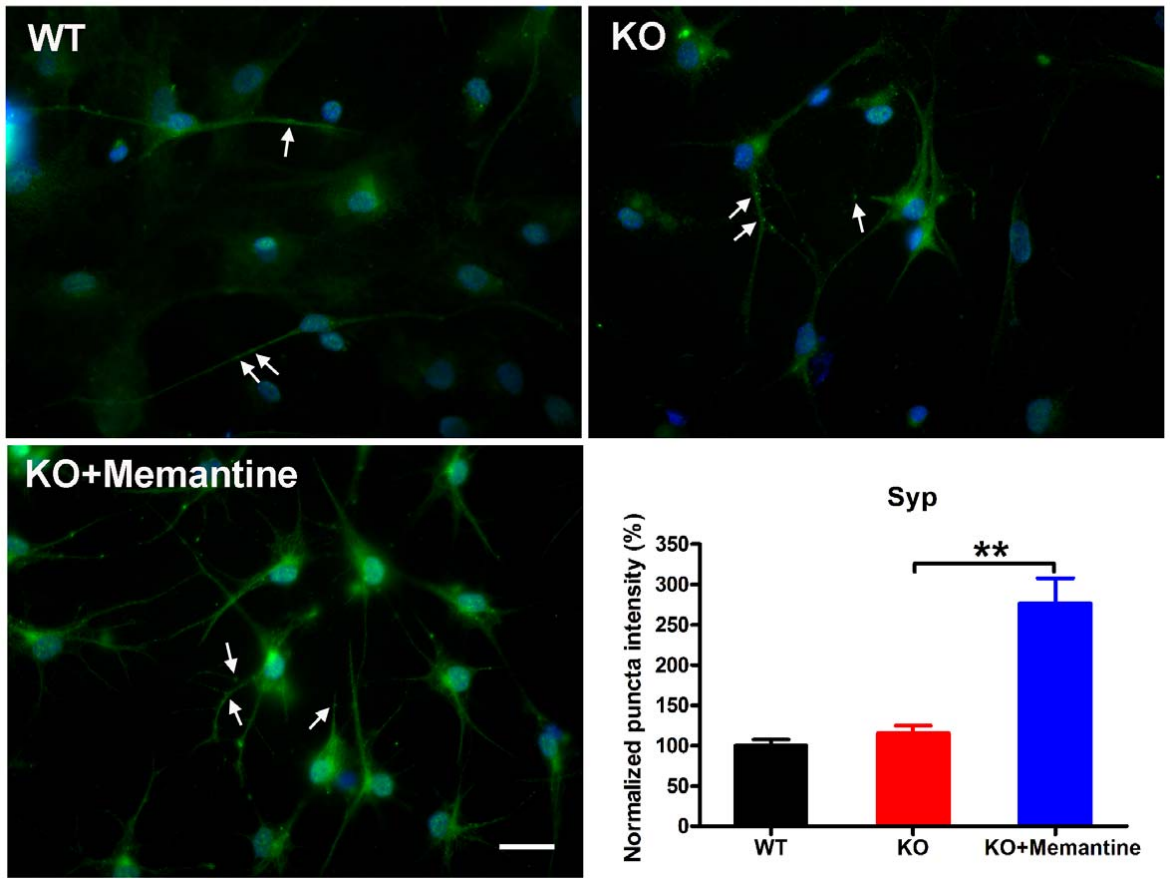
Memantine stimulated the formation of total synapses in *Fmr1*-KO CGCs. Representative photomicrographs of labeling of Syp (arrow) in WT CGCs, *Fmr1*-KO CGCs and *Fmr1*-KO CGCs treated with memantine. Green, Syp; Blue, Hoechst, scale bars, 25 µm. The histogram shows quantification of puncta size using image analysis. WT, n = 23; *Fmr1*-KO, n = 25; *Fmr1*-KO+Memantine, n = 26. ^**^p<0.01.

**Figure 7:**
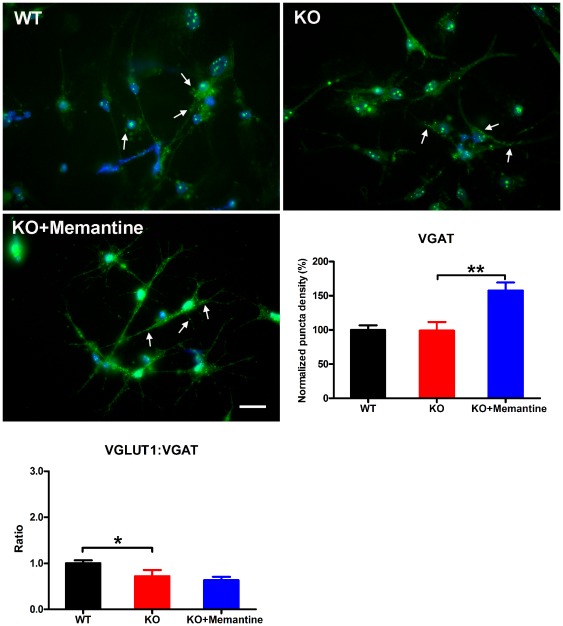
Memantine stimulated the formation of inhibitory synapses in *Fmr1*-KO CGCs. Representative photomicrographs of labeling of VGAT (arrow) in WT CGCs, KO CGCs and KO CGCs treated with memantine. Green, VGAT; Blue, Hoechst, scale bars, 25 µm. The histogram shows quantification of puncta size using image analysis and ratio of excitatory synapses to inhibitory synapse. WT, n = 23; *Fmr1*-KO, n = 25; *Fmr1*-KO+Memantine, n = 25. ^*^p<0.05, ^**^p<0.01.

To further investigate the effects of memantine on synapse formation, using antibodies to synaptic vesicle proteins we examined synapse formation comparing WT CGCs, *Fmr1*-KO CGCs and *Fmr1*-KO CGCs treated with memantine. We stained the CGCs with antibodies to synaptophysin (a general marker of all synapses) and to the vesicular glutamate transporter VGLUT1 and the vesicular GABA transporter VGAT (markers of excitatory and inhibitory synapses respectively). We observed a dramatic decrease in the immunoreactivity of excitatory synapses (VGLUT1 staining) in *Fmr1*-KO CGCs as compared with that in WT CGCs (one-way ANOVA, F = 5.44, p = 0.01; Fisher's PLSD, p = 0.01, Figure 5). Memantine treatment significantly stimulated excitatory synapse formation in *Fmr1*-KO CGCs (Figure 5, Fisher's PLSD, p = 0.01) and fully restored the intensity of excitatory synapse to the level observed in WT CGCs (Figure 5). In addition, we found that the intensities of total synapses (Syp staining) and inhibitory synapses (VGAT staining) were not altered in *Fmr1*-KO CGCs as compared with that in WT CGCs (one-way ANOVA, F = 25.52, p<0.0001; Fisher's PLSD, p = 0.58, Figure 6 and one-way ANOVA, F = 10.53, p = 0.001; Fisher's PLSD, p = 0.95, Figure 7, respectively). Memantine treatment also stimulated the formation of total synapses and inhibitory synapses in *Fmr1*-KO CGCs (Fisher's PLSD, p<0.0001, Figure 6 and p = 0.001, Figure 7, respectively). The ratio of excitatory to inhibitory synapses in *Fmr1*-KO CGCs was lower than in WT CGCs (one-way ANOVA, F = 4.70, p = 0.02; Fisher's PLSD, p = 0.04). However, memantine treatment did not change the ratio in *Fmr1*-KO CGCs (Fisher's PLSD, p = 0.52, Figure 7).

## Discussion

The hypo-function of the GABAergic system and the glutamate toxicity found in autism have been implicated as possibly having a causal role for autism [5], [20]. Memantine is an uncompetitive antagonist at NMDA receptors [11], [12] and is widely used for the treatment of AD. Memantine has been demonstrated to have neuroprotective effects and promote neurogenesis [43], [44]. Most recently, several clinical trials have reported that memantine can significantly improve language function and social behavior of patients with autism and fragile X syndrome (FXS) [24], [25], [26], [27]. Neuroanatomical studies on autistic individuals have revealed loss of purkinje and granule cells of the cerebellum [45], [46] and cerebellar dysfunction in animal models can induce autism-like behavioral deficits [47], [48]. Granule cells of the cerebellum constitute the largest homogeneous neuronal population of mammalian brain. Due to their postnatal generation and the feasibility of well characterized primary in vitro cultures, cerebellar granule cells (CGCs) are a model of choice for the study of neural development, function and pathology [49]. In this study, using cultured CGCs from *Fmr1*-KO, we first examined how memantine affects the cell properties. We found that memantine significantly promoted the adhesion of CGCs, while having little effect on the cell migration.

Integrin-mediated cell adhesion has been shown to be essential in the regulation of neuronal migration, which is a critical process that determines the final location of neurons, and thus establishes the basis for neural circuits [40], [50], [51], [52]. In addition, the complexity and specificity of synaptogenesis relies upon regulation of cell adhesion molecules that mediate contact initiation, synapse formation and plasticity. Disruption of adhesion could result in an imbalance in the structure and function of synapses [53], [54]. Recently, several lines of studies have shown that mutations in the synaptic adhesion molecules Neurexin 1 and Neuroligins 3 and 4 are associated with autism [54], [55]. Mutations in scaffolding molecule Shank3 that interacts with Neuroligins have also been detected in some autistic subjects [56], [57]. These observations imply that a defect in synaptic cell adhesion may alter synapse formation, maturation and plasticity in autistic subjects. Since memantine promotes CGC adhesion, we reason that it could play a role in the regulation of neural synaptogenesis. Thus, memantine might counteract some of the defects in synaptic cell adhesion molecules found in autism. In our study, we found that the adhesion of *Fmr1*-KO CGCs was increased as compared with controls. Whether there is a defect in adhesion-associated molecules in *Fmr1*-KO mice or FXS patients is unknown and our results suggest that it is of important significance to further investigate synaptic cell adhesion and migration properties in FXS in the future.

Our study further demonstrated that memantine stimulated synapse formation. In particular, memantine restored the number of excitatory synapses found in *Fmr1*-KO CGCs to the level of that in the WT CGCs. Synapses critically mediate neuronal communication. The number, type, and connectivity patterns of synapses determine the formation, maintenance, and function of neural circuitries [58]. Recently, Yun and Trommer [59] reported that that the peak amplitude of NMDA receptor-mediated excitatory postsynaptic currents (EPSCs) was smaller in *Fmr1*-KO mice as compared with controls, while AMPA receptor-mediated EPSCs were comparable in the two groups. In addition, they found diminished medial perforant path-granule cell long-term potentiation (LTP), complementing previous findings that demonstrated impaired LTP in CA1, neocortex, and amygdala and exaggerated long-term depression in CA1 of *Fmr1*-KO mice [60]. We suggest that the stimulatory effect of memantine on excitatory synapse formation could counter the smaller EPSCs found in *Fmr1*-KO CGCs and exert a therapeutic role. However whether memantine can actually promote excitatory synaptic transmission remains to be further studied.

In addition, we found that memantine also stimulated the formation of inhibitory synapses. Thus memantine treatment did not change the ratio of excitatory to inhibitory synapses. This leaves open the question of whether memantine affects the neural circuit balance. Future studies will be needed to answer this question. The stimulatory effect of memantine on both excitatory and inhibitory synapse formation suggests that memantine may have a general role in promoting neurogenesis. Recently, several studies have shown that memantine promotes cell proliferation and production of mature granule neurons in the adult hippocampus [43], [61]. Memantine has also been demonstrated to stimulate the proliferation of hippocampal progenitor cells [62]. All these findings imply that memantine can play a role in neurogenesis.

To further elucidate the role of memantine on neural development and function, we examined dendritic spines. We showed that memantine treatment promotes the development of mature (mushroom-shaped) dendritic spines and can restore the dendritic spine to normal levels in *Fmr1*-KO CGCs. Several categories of spines have been identified based on their shape and size, including thin, stubby, cup, and mushroom shaped [63]. Spine morphology is linked to synapse function and the mushroom-shaped spines considered to represent the most mature and stable spine morphology [36], [64]. Recent studies suggest that excitatory synapses mainly connect to mushroom-shaped dendritic spines [65]. Thus, the increase in mushroom-shaped spines in response to memantine treatment is consistent with our findings that memantine stimulates the formation of excitatory synapses. The effect of memantine on spines in *Fmr1*-KO CGCs also suggests a role of memantine in restoring synaptic transmission.

A number of studies have reported defects of dendritic spines in *Fmr1*-KO mice [38], [66]. It has been reported that the length of dendritic spines, a measure of immaturity, is increased in *Fmr1*-KO mice, suggesting a delay in the development of dendritic spines. In our study, we found that the length of dendritic spines was significantly impaired in *Fmr1*-KO CGCs, which is supportive of the findings from Nimchinsky et al [66]. The effect of memantine in enhancing dendritic spine maturation could contribute to its observed therapeutic effects in autistic and FXS patients. We reckon there could be two ways memantine exert its restoring role in FMRP deficient neurons. It could specifically counteract the loss of FMRP and therefore only acting on those dendritic spines that are immature as a result of FMRP loss. Alternatively memantine could have a more general effect on the development and maturation of dendritic spines and not directly target those neurons altered by the loss of FMRP. Studies will be carried out on wild type mice to further elucidate the mechanism through which memantine affects the neuronal properties, synapse formation and dendritic spine maturation.

In summary, we have shown that *Fmr1*-KO CGCs exhibit delayed development of dendritic spines and reduced excitatory synapse formation. Memantine treatment of *Fmr1*-KO CGCs promoted cell adhesion. Memantine also stimulated the development of mushroom-shaped dendritic spines and restored the dendritic spine to normal levels in *Fmr1*-KO CGCs. Furthermore, we demonstrated that memantine treatment can enhance synapse formation, particularly excitatory synapses and it restored the excitatory synapses to a normal range in *Fmr1*-KO CGCs. These findings suggest that the stimulatory effect of memantine on dendritic spines development and excitatory synapse formation, as well as the adhesion promoting effect could be mechanisms by which memantine exerts its therapeutic effect in autism and FXS.
